# Traumatic Aortic Injury: Sailing Close to the Wind

**DOI:** 10.7759/cureus.20264

**Published:** 2021-12-08

**Authors:** Rajeev Thilak Chellasamy, Srujana Reddy, Saichandran B V, Rajkumar Sundararaj

**Affiliations:** 1 General Surgery, Madras Medical College, Chennai, IND; 2 Cardiothoracic and Vascular Surgery, Jawaharlal Institute of Postgraduate Medical Education and Research, Puducherry, IND; 3 Cardiothoracic Surgery, Jawaharlal Institute of Postgraduate Medical Education and Research, Puducherry, IND; 4 Anesthesiology, Jawaharlal Institute of Postgraduate Medical Education and Research, Puducherry, IND

**Keywords:** emergency medical service, major trauma, aorta injury, thoracic aorta, road traffic injuries

## Abstract

Blunt aortic injuries are lethal and only a few patients survive. Most of the patients die at the site of accidents and only a few reach the hospital. Those who reach hospitals usually have small tears or pseudo-aneurysm of the aorta. Immediate imaging and intervention play a major role in the survival of these patients. We report this case as only a few patients report to the hospital with aortic injury and our patient was taken up for surgery immediately and a life-saving procedure was done.

## Introduction

Trauma is one of the leading causes of death in the world. Blunt aortic injury is lethal and it accounts for about 18% of all road traffic accident-related deaths. Most of the patients die immediately and 50% of those who survive, die within 24 hours if left untreated [1]. Most of the survivors, who arrive at the emergency department, have small pseudo-aneurysm or partial-thickness tears. Rapid transport, resuscitation, and immediate intervention can improve the survival of patients with aortic injury [2]. We report a 50-year-old male patient who reported to the emergency department with an alleged history of road traffic accidents. He presented with hypotension and was resuscitated. On evaluation, he was found to have an injury of the proximal part of descending thoracic aorta and the proximal segment of the left subclavian artery. He was taken for operation immediately. The injured part of the aorta was replaced with an interposition woven polyester graft and the left carotid to subclavian artery bypass was done with a saphenous graft. His postoperative course was uneventful. Aortic injuries are dreadful, requiring immediate resuscitation, evaluation, and management for survival. Some of the cases of aortic injuries are also manageable with endovascular procedures depending upon the location and site of injury and availability of the facilities and expertise. In our case of aortic injury, as the patient was hemodynamically unstable and also associated with the involvement of the subclavian artery, we preferred to manage by surgical intervention. 

## Case presentation

**Figure 1:**
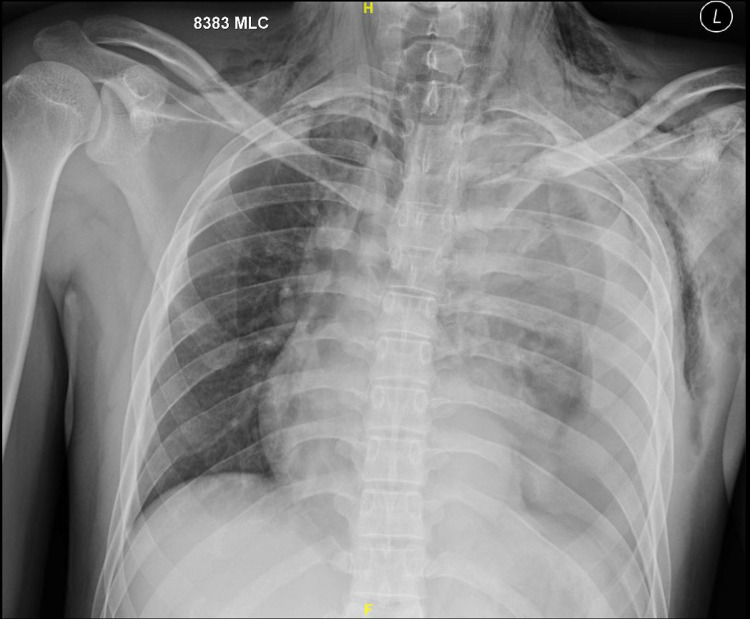
Chest X-ray showing left-sided hemo-thorax with left clavicle, left first, second, and third rib fracture.

**Figure 2:**
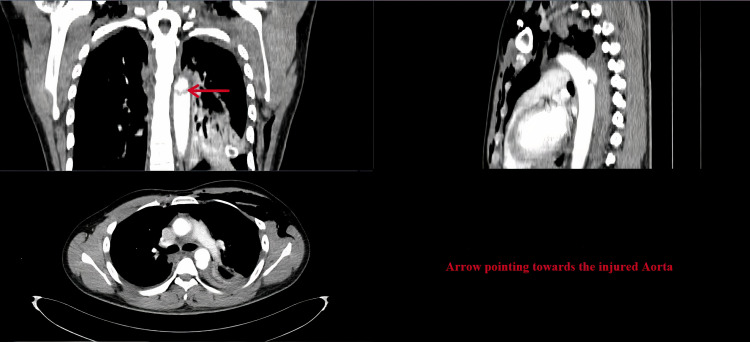
CT angiogram showing injured aorta.

A 50-year-old male patient had a blunt injury to his thorax and was admitted to the emergency department. He presented with hypotension on admission. He was conscious and tachypneic. He was resuscitated immediately with IV fluid. Chest X-ray showed massive hemothorax with the left side first, second, third rib, and left clavicle fracture (Figure 1). An intercostal drainage (ICD) tube was placed. ICD was clamped immediately as it drained 1.5 L of blood immediately. Contrast-enhanced computed tomography (CECT) thorax was done. It showed proximal descending aortic injury with contusion of the proximal part of the left subclavian artery (Figure 2). Since his hemodynamic was unstable he was taken for surgery immediately.

**Figure 3:**
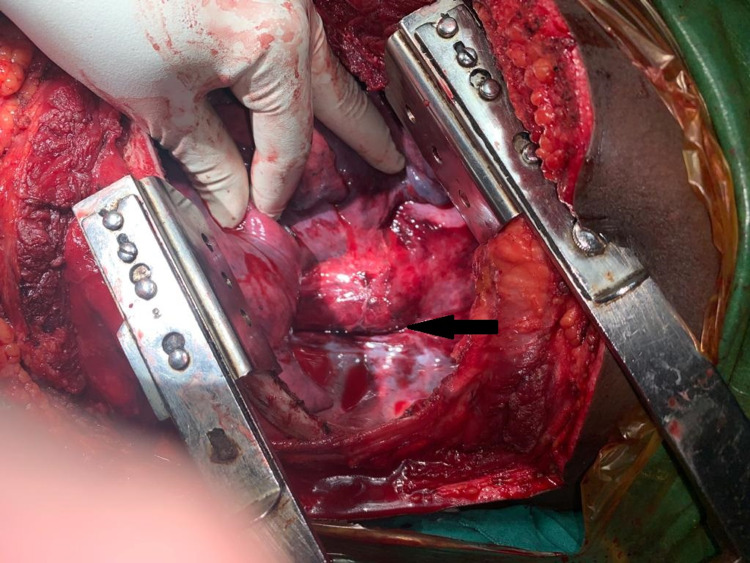
Black arrow showing injured aorta.

**Figure 4:**
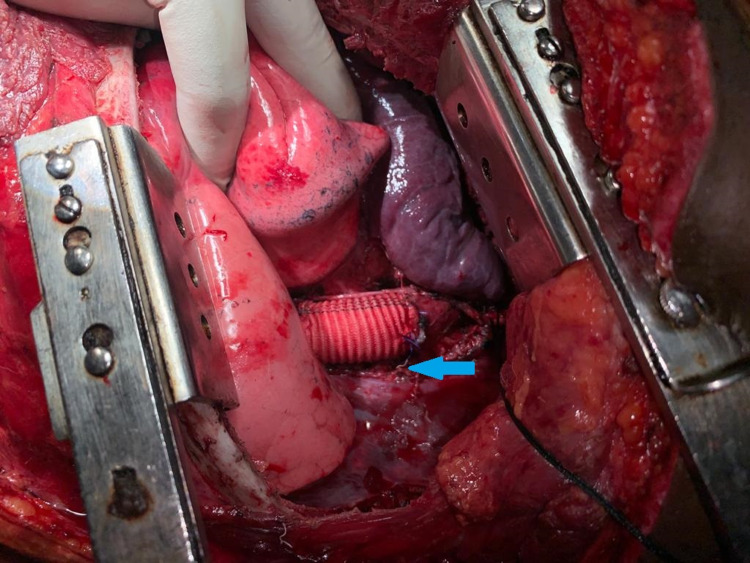
Blue arrow showing injured aorta replaced with Dacron graft.

**Figure 5:**
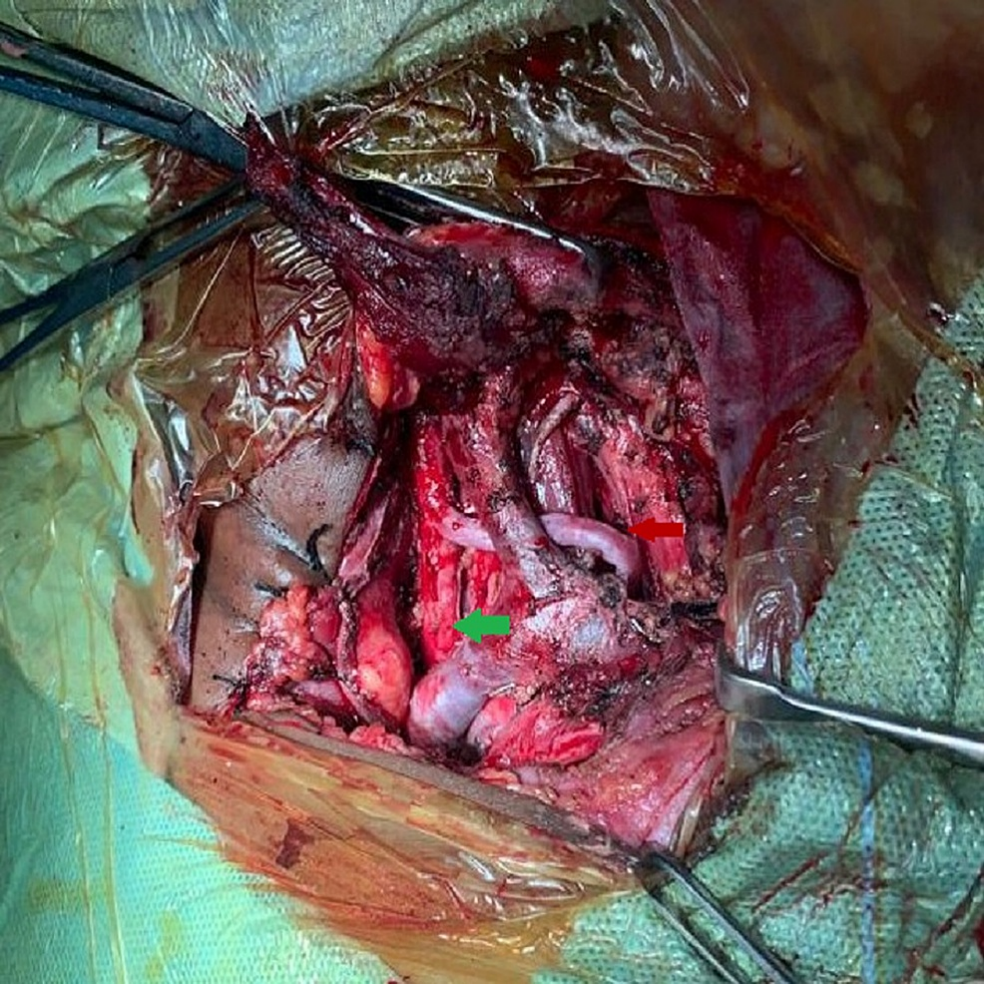
Green arrow pointing towards left common carotid and red arrow pointing towards saphaneous vein graft.

A double-lumen endotracheal tube was placed. The chest was opened through the left postero-lateral incision. The left lung was collapsed and descending thoracic aorta (DTA) was approached. The left subclavian artery was dissected from the origin and the injured part was identified. The left subclavian artery (SCA) was looped with thick silk proximally. Proximal and distal clamps were applied on DTA isolating the injured part. The injured part of DTA was excised and replaced with a Dacron graft (Figures 3-4). Then, the proximal left subclavian artery was ligated with a silk suture. The thoracotomy wound was closed. An incision was placed over the neck. The left common carotid artery was dissected out from the carotid sheath. The left subclavian artery was traced and identified. Branches of the first part of the left subclavian arteries were preserved. Left common carotid to left subclavian bypass was done using a venous graft (Figure 5). The patient was shifted to the intensive care unit (ICU) after the procedure. The postoperative course was uneventful. A post-operative CT angiogram was done which showed an intact aorta. He followed up after one month and again at six months. He had no complaints.

## Discussion

The first report on the traumatic rupture of the thoracic aorta was made by Vesalius in 1557. Aortic injury is the second common cause of death following road traffic accidents. Most of the patients die at the site of the accident. Only 15% of the patients arrive at the hospital. Some 30% of the survivors usually die within six hours while another 20% by 24 hours if the diagnosis is delayed. Most of the patients come with a partial tear or pseudo-aneurysm with intact adventitia though there is a risk of complete rupture. Complete disruption of the aorta is always fatal [1-3]. One study revealed that 12.7% of injury-related deaths are due to blunt and penetrating injuries of the thoracic aorta and aortic arch branches. The initial stage of aortic injury starts with pseudo-aneurysm which can grow slowly or rapidly. They can remain intact for several days or months. In the final stages, they rupture and it may lead to massive hemorrhage and eventually death. Our case presented with partial tear with pseudo-aneurysm [1]. The proximal part of the descending thoracic aorta, otherwise known as the isthmus is the most common segment to be involved. Although any part of the aorta can be injured in blunt trauma. The arch of the aorta is mobile whereas the descending thoracic aorta is immobile. The arch of the aorta suddenly moves away from the descending aorta during sudden deceleration making the isthmus prone to injury. Eighty percent of aortic injury involves the isthmus. Our patient had an injury at the same site following a road traffic accident [4-5].

Most of the patients present with vague symptoms which include difficulty in swallowing, speaking, hoarseness of voice, atypical chest pain, or shortness of breath. Clinical signs include stridor, sudden onset of upper extremity hypertension with the difference in pulse volume between upper and lower extremities, or harsh systolic murmur over the precordium or inter-scapular area. Mechanism of injury, imaging, and clinical signs aid in diagnosing an aortic injury.

Chest X-ray is the first investigation taken which has a high negative predictive value of about 98%. A clear aortic outline from the aortic arch to the diaphragm ensures that there is no aortic injury [6]. Mediastinal hemorrhage resulting from tears of mediastinal vascular structures may be indicative of an aortic injury. A mediastinal width of more than 8 cm at the level of the aortic arch is an indicator for further assessment of the aortic injury. Widened mediastinum has an 83% positive predictive value for aortic trauma. Apical pleural cap, deviation of a nasogastric tube to right, depression of the left main bronchi are a few other imaging signs that may be indicative of aortic injury. But these are less sensitive [6-8].

Angiography was the gold standard for screening of aortic injury in the past, which has been replaced by a CT angiogram (CTA). CTA can be done rapidly. Angiography is only indicated when CT is inconclusive. The sensitivity of the CT scan is around 97%-100%. Pseudo-aneurysm or intimal flap is a classical finding in CT scans. The immediate intervention has to be planned if uncontained extravasation of contrast is observed while doing a CT. Other features include displacement of the trachea and esophagus [9-10].

Aortic injuries can be classified into three categories [11]. The first category includes contained aortic injuries. Patients can be managed conservatively with blood pressure control. Patients’ hemodynamics can be unstable due to associated injuries. They may present with other injuries such as splenic injury or pelvic injury where hemorrhage control is of prime importance. Naso-gastric tube insertion and endotracheal tube placement should be placed with caution. The natural history of the patients who were managed conservatively is unknown. Most aortic injuries require intervention eventually [12]. Some of the relative contraindications for immediate repair are severe head injury, pulmonary injury, coagulopathy disorder, acidosis, hypothermia, and patients with severe co-morbidities [13]. Initial management of the contained aortic injury is to prevent any rupture. Overcorrection with fluids is generally avoided as there is a risk of rupture in contained aortic injury. Ideal systolic pressure has to be maintained around 120 whereas few suggest 90 when there is no head injury. The patient can be started on short-acting anti-hypertensives [13].

The second type includes those patients who are hemodynamically unstable due to aortic injury or associated injuries. These patients require immediate intervention. Our patient was hemodynamically unstable and was taken for immediate surgery. The third type includes those patients who had complete aortic transection. They usually die at the accident site.

Patients can be managed by surgery or by endovascular technique. The endovascular procedure requires expertise and the availability of stents. Not all patients can be subjected to stent placement. Mortality with surgery is around 24% and the risk of paraplegia is around 19% [14]. Surgery offers a complete resolution of the problem and offers better long-term survival benefits. In our case, the patient had unstable hemodynamics and the landing zone for stent placement was difficult as the proximal part of the left subclavian artery was contused. He underwent a successful surgical repair and was discharged without any complications.

## Conclusions

Aortic injury is lethal and needs urgent evaluation. Cautious resuscitation, immediate imaging, and planning play a crucial role in the management of aortic injury. Management of aortic injury includes surgery or endovascular stent placement. Only a few patients can be subjected to stents and the availability of stents also has a major role in management. Surgery offers a long-term solution. Surgery is always advised for unstable patients as in our case.
